# Glyoxylic Acid, an α-Keto Acid Metabolite Derived from Glycine, Promotes Myogenesis in C2C12 Cells

**DOI:** 10.3390/nu15071763

**Published:** 2023-04-04

**Authors:** Toshio Norikura, Yutaro Sasaki, Akiko Kojima-Yuasa, Atsushi Kon

**Affiliations:** 1 Department of Nutrition, Faculty of Health Science, Aomori University of Health and Welfare, Aomori 030-8505, Japan; 2 Department of Nutrition, Graduate School of Human Life and Ecology, Osaka Metropolitan University, Osaka 558-8585, Japan

**Keywords:** α-keto acid, glyoxylic acid, C2C12 cells, myogenesis, muscle atrophy

## Abstract

α-Keto acids may help prevent malnutrition in patients with chronic kidney disease (CKD), who consume protein-restricted diets, because they serve as amino acid sources without producing nitrogenous waste compounds. However, the physiological roles of α-keto acids, especially those derived from non-essential amino acids, remain unclear. In this study, we examined the effect of glyoxylic acid (GA), an α-keto acid metabolite derived from glycine, on myogenesis in C2C12 cells. Differentiation and mitochondrial biogenesis were used as myogenesis indicators. Treatment with GA for 6 d resulted in an increase in the expression of differentiation markers (myosin heavy chain II and myogenic regulatory factors), mitochondrial biogenesis, and intracellular amounts of amino acids (glycine, serine, and alanine) and their metabolites (citric acid and succinic acid). In addition, GA treatment suppressed the 2.5-µM dexamethasone (Dex)-induced increase in mRNA levels of ubiquitin ligases (*Trim63* and *Fbxo32*), muscle atrophy markers. These results indicate that GA promotes myogenesis, suppresses Dex-induced muscle atrophy, and is metabolized to amino acids in muscle cells. Although further in vivo experiments are needed, GA may be a beneficial nutrient for ameliorating the loss of muscle mass, strength, and function in patients with CKD on a strict dietary protein restriction.

## 1. Introduction

Age-related loss of muscle mass, strength, and function, referred to as sarcopenia, is associated with fall-related fractures, functional decline, and hospitalization, leading to higher rates of mortality and morbidity in elderly individuals [1]. The rate of muscle protein synthesis is regulated by responses to anabolic stimuli, such as food intake and physical activity [2]. Delayed amino acid absorption and protein anabolic resistance are common in elderly individuals [3,4]. Therefore, the elderly require more dietary protein to increase the rate of muscle protein synthesis.

Aging is associated with gradual changes in kidney structure and function, even in the absence of chronic diseases. Chronic kidney disease (CKD) is characterized by excessive accumulation of uremic toxins, urea, and other nitrogenous waste compounds that are not eliminated owing to a decline in renal function. Most uremic toxins derived from dietary proteins accelerate CKD progression [5]. Therefore, dietary protein restriction is recommended to slow CKD progression; however, it may lead to sarcopenia progression, making sarcopenia a major complication of CKD [6]. The Kidney Disease Outcomes Quality Initiative (KDOQI) guidelines, updated in 2020, propose supplementation of nitrogen-free α-keto acids to reduce the risk of malnutrition in patients with CKD whose diets are protein-restricted [7]. This is because α-keto acids serve as a source of amino acids without producing nitrogenous waste compounds derived from the amino group. However, to the best of our knowledge, keto acids have reportedly been used only as a source of essential amino acids.

Glyoxylic acid (GA), an α-keto acid metabolite of glycine, is produced in the body as a by-product of the pentose phosphate pathway and as a breakdown product of serine and hydroxyproline [8]. It is a precursor of oxalate, whose dietary intake and endogenous synthesis are associated with urinary stones. Ascorbic acid is the primary dietary precursor in the endogenous production of oxalate [9]. Glycine is synthesized primarily from serine, threonine (present in most mammals but absent in humans) [10], choline, and GA; however, the level synthesized is insufficient under certain conditions [8]. Indeed, supplemental glycine protects muscle mass and function in wasting models, such as cancer cachexia [11] and sepsis models [12], and under calorie-restricted conditions [13]. Therefore, GA may play a greater role as a source of glycine than oxalic acid under certain conditions. In addition, GA inhibits the proliferation of human colon cancer cells [14]. In this milieu, we hypothesized that GA may act as a bioactive substance or a source of glycine in skeletal muscle.

A decline in differentiation capacity and mitochondrial biogenesis is a typical feature of age-related muscular dysfunction in myogenesis [15,16]. To the best of our knowledge, no study has reported the effect of GA on skeletal muscles. Elucidating the physiological effects of GA may contribute to the development of new dietary therapies to prevent and ameliorate sarcopenia in patients with restricted protein intake. To address this hypothesis, we examined the effect of GA on differentiation and mitochondrial biogenesis in C2C12 cells, a well-established model for studying myogenesis, a crucial process regulating skeletal muscle regeneration and homeostasis.

## 2. Materials and Methods

### 2.1. Materials

The mouse skeletal muscle cell line C2C12 was provided by the Riken Cell Bank (Ibaraki, Japan). The rat skeletal muscle cell line L6 was provided by the JCRB Cell Bank (Osaka, Japan). DMEM, neutral red, and GA were purchased from Fujifilm Wako Pure Chemical Co. (Osaka, Japan). Rhodamine 123 was purchased from Dojindo. Laboratories (Kumamoto, Japan).

### 2.2. Cell Culture and Treatment

**Figure 1:**
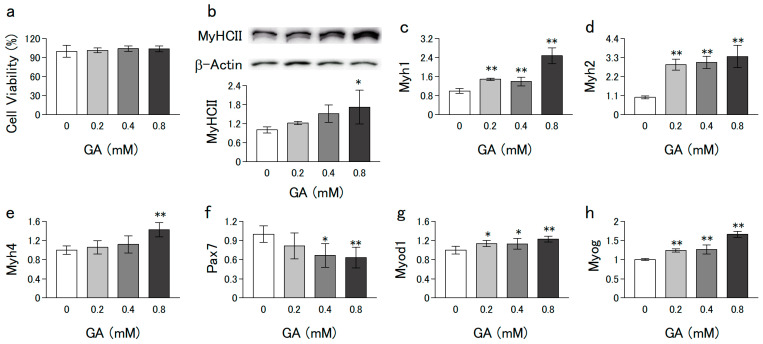
Effect of GA on the viability and differentiation of C2C12 cells. C2C12 cells grown to subconfluence in a growth medium (without GA) were further cultured in a differentiation medium with or without GA for 6 d. (**a**) Cell viability was measured using the neutral red assay. (**b**) Western blot images of MyHC II, a biomarker protein for differentiation, and β-actin, an internal control. (**c**–**e**) mRNA levels of MyHCII encoding isoforms (*Myh1*, *Myh2*, and *Myh4*). (**f**) mRNA levels of *Pax7*, a satellite cell marker. (**g**,**h**) mRNA levels of myogenic regulatory factors (*Myod1* and *Myog*). mRNA levels were measured using qPCR. Values are represented as mean ± SD of three independent experiments. Data were analyzed using a one-way analysis of variance followed by Dunnett’s post hoc comparison tests. The mean value was significantly different from that of the control: ** *p* < 0.01, * *p* < 0.05.

**Figure 2:**
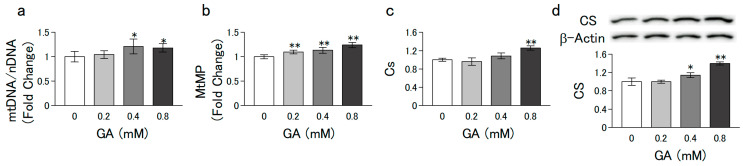
Effect of GA on mitochondrial biogenesis in C2C12 cells. C2C12 cells grown to subconfluence in a growth medium (without GA) were further cultured in differentiation medium with or without GA for 6 d. (**a**) Relative mtDNA copy number measured using qPCR. Results are shown as fold change values compared with the results of the control group. (**b**) MtMP measured via Rho123 uptake. Results are shown as fold change values compared with the results of the control group. (**c**) mRNA levels of *Cs*. The mRNA levels were measured using qPCR. (**d**) Western blot images of CS and β-actin, the internal control. Values are presented as mean ± SD of three independent experiments. Data were analyzed using a one-way ANOVA followed by Dunnett’s post hoc comparison tests. Mean value was significantly different from that of the control: ** *p* < 0.01, * *p* < 0.05.

**Figure 3:**
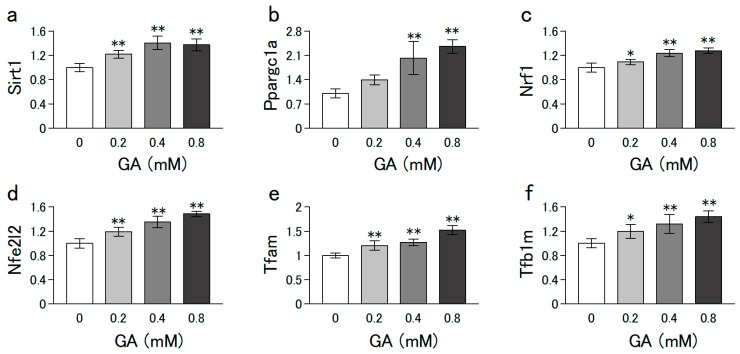
Effect of GA on the levels of mRNAs involved in mitochondrial biogenesis in C2C12 cells. C2C12 cells grown to subconfluence in a growth medium (without GA) were further cultured in a differentiation medium with or without GA for 6 d. mRNA levels of (**a**) Sirt1, (**b**) Ppargc1a, (**c**) Nrf1, (**d**) Nfe2l2 (**e**) Tfam (**f**) Tfb1m were measured using qPCR. Values are presented as mean ± SD of three independent experiments. Data were analyzed using a one-way ANOVA followed by Dunnett’s post hoc comparison tests. Mean value was significantly different from that of the control: ** *p* < 0.01, * *p* < 0.05.

**Figure 4:**
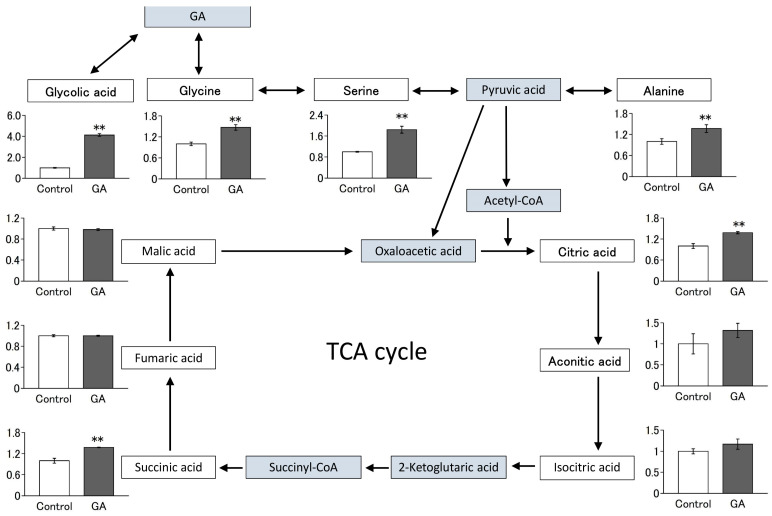
Changes in the intracellular content of several amino acids and their metabolites caused by GA. C2C12 cells grown to subconfluence in a growth medium (without GA) were further cultured in a differentiation medium with or without GA for 6 d. Relative metabolite changes shown in the graphs were obtained using GC-MS analysis. Values are presented as mean ± SD of three independent experiments. Statistical differences were determined using Student’s *t*-test. Differences with ** *p* < 0.01 was considered significant.

**Figure 5:**
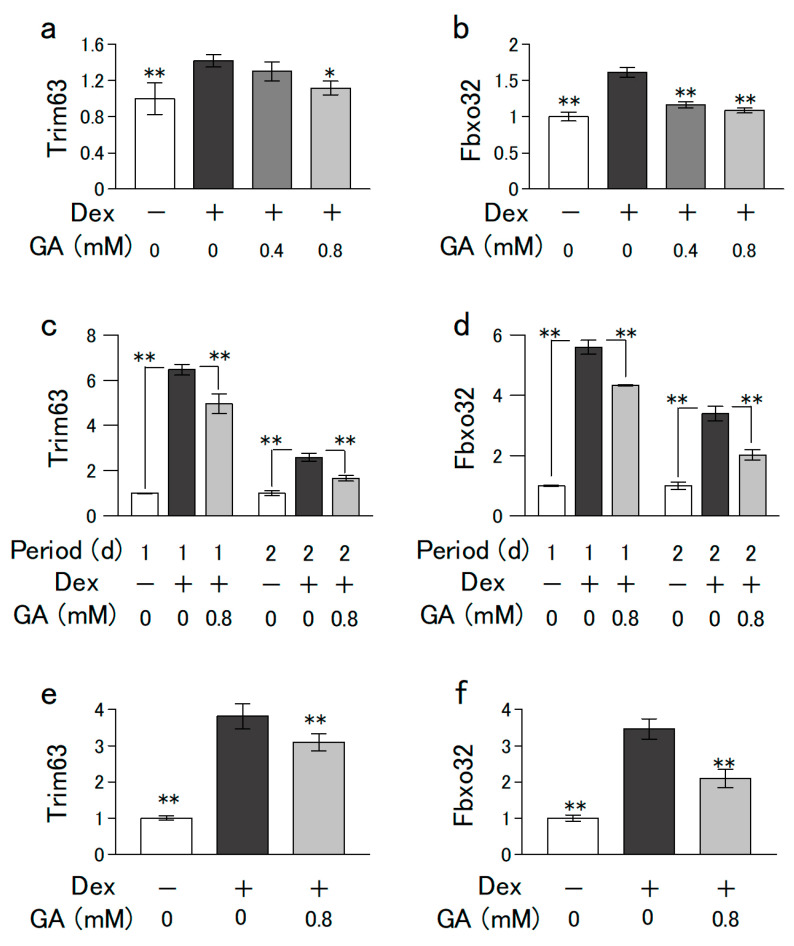
Effect of GA and Dex on the mRNA expression levels involved in muscle atrophy in C2C12 cells. (**a**,**b**) C2C12 cells grown to subconfluence in a growth medium (without GA) were further cultured in a differentiation medium without GA for 6 d. The cells were further cultured for 1 d in a differentiation medium with or without Dex (2.5 µM) and GA (0–0.8 mM). (**c**,**d**) C2C12 cells grown to subconfluence in a growth medium (without GA) were further cultured for the indicated days in a differentiation medium with or without Dex (2.5 µM) and GA (0.8 mM). (**e**,**f**) L6 cells grown to subconfluence in a growth medium (without GA) were further cultured in a differentiation medium without GA for 6 d. The cells were further cultured for 1 d in a differentiation medium with or without Dex (2.5 µM) and GA (0.8 mM). mRNA levels of ubiquitin ligases (Trim63 and Fbxo32), markers of muscle atrophy, were measured using qPCR. Values are presented as mean ± SD of three independent experiments. (**a**,**b**,**e**,**f**) Data were analyzed using a one-way ANOVA followed by Dunnett’s post hoc comparison test. Mean value was significantly different from that of Dex: (**c**,**d**) Data were analyzed using a one-way ANOVA followed by Tukey post hoc comparison test. ** *p* < 0.01, * *p* < 0.05.

C2C12 and L6 cells were grown in growth medium (low-glucose DMEM supplemented with 10% FBS, 100 U/mL penicillin, and 100 ng/mL streptomycin) at 37 °C in a 5% CO_2_ humidified incubator. The cells (5 × 10^5^/ϕ 35-mm dish) were proliferated to subconfluence by preculturing for 4 d. In the experiments of Figure 1, Figure 2, Figure 3 and Figure 4 thereafter, the medium was changed to a differentiation medium (DMEM containing 2% horse serum) with or without different concentrations of GA (0–0.8 mM) for 6 d. In the experiment of Figure 5a,b,e,f, the cells that differentiated after 6 d of culturing (without GA) were further cultured for 1 d in medium with or without dexamethasone (Dex) and GA. In the experiment of Figure 5c,d, C2C12 cells grown to subconfluence in a growth medium (without GA) were further cultured for indicated days in a differentiation medium with or without Dex (2.5 µM) and GA (0.8 mM).

### 2.3. Cell Viability

Cell viability was examined using the neutral red assay, as described previously [17]. After 6 d of incubation, the cell culture medium was replaced with a neutral red solution (0.005%) in PBS. Following incubation for 30 min, intracellular neutral red was extracted using a mixture of 1% acetic acid and 50% ethanol, and the absorbance of each sample was measured at 540 nm using a microplate reader.

### 2.4. Western Blot Analysis

The C2C12 cells were harvested and lysed using RIPA buffer containing protease and phosphatase inhibitor cocktails (Nacalai Tesque, Kyoto, Japan). Equal amounts of protein were separated by SDS-PAGE and transferred to PVDF membranes. After overnight blocking with 1% non-fat milk powder dissolved in TBS-T, the membranes were incubated with anti-MyHCII (R&D Systems Inc., Minneapolis, MN, USA), citrate synthase (CS), and anti-β-actin (Cell Signaling Technology, Danvers, MA, USA) antibodies for 1 h at room temperature. After several washes, the membranes were incubated with HRP-conjugated secondary antibody (Cell Signaling Technology) for 1 h at room temperature. Finally, the bands were developed using EzWestLumi Plus (Atto, Tokyo, Japan).

### 2.5. Real-Time PCR

The total RNA was isolated from C2C12 cells using a QuickGene RNA cultured cell kit S (Kurabo, Osaka, Japan) and reverse-transcribed to cDNA using the ReverTra Ace qPCR RT Master Mix (Toyobo, Osaka, Japan) according to the manufacturer’s instructions. Quantitative real-time PCR was performed using Thunderbird SYBR qPCR Mix (Toyobo) with StepOnePlus (Thermo Fisher Scientific, Waltham, MA, USA). Relative mRNA expression levels were calculated using the ΔΔCt analysis and normalized to the level of the internal control (*Actb*). The primer sequences used are listed in Supplementary Table S1. Melt curve analysis results of the qPCR products are shown in Supplementary Figure S1.

### 2.6. Mitochondrial DNA Copy Number

To determine the relative mitochondrial DNA (mtDNA) copy number, nuclear DNA (nDNA) and mtDNA were isolated using a NucleoSpin Tissue Kit (Takara, Kyoto, Japan). The relative mtDNA copy number was calculated from the ratio of the mitochondrial gene copy number (16S rRNA gene (16S)) to nuclear gene copy number (β-actin gene (Actb)) quantified using real-time PCR (ΔΔCt analysis), as described above.

### 2.7. Mitochondrial Membrane Potential

Mitochondrial membrane potential (MtMP) was measured using a cell-permeant, cationic green fluorescent dye, rhodamine 123, according to a previously reported method [18], with minor modifications. Briefly, after 6 d of incubation, the C2C12 cells were incubated with rhodamine 123 (10 µg/mL) and with or without GA in DMEM for 30 min. Next, rhodamine 123 uptake was terminated by washing the cells with PBS. Thereafter, the cells were lysed using 1% Triton X-100, and the fluorescence intensity (485 nm/520 nm) was measured using a fluorescence microplate reader.

### 2.8. Metabolome Analysis

After 6 d of incubation, the C2C12 cells were washed twice with PBS and then harvested using cold (–30 °C) methanol containing 2-isopropylmalic acid as the internal standard. After freezing and thawing, the cell suspension was centrifuged at 20,000× *g* for 15 min at 4 °C. Subsequently, the supernatant and GC-MS standard mixture containing 52 metabolites (GL Science, Tokyo, Japan) were dried using a centrifugal evaporator and subjected to methoxymization and trimethylsilyl derivatization according to the manufacturer’s instructions. We used the QP2010SE system equipped with an AOC-20i autosampler (Shimadzu, Kyoto, Japan) and an Inert Cap 5MS/NP capillary column (30 m × 0.25 mm × 0.25 µm, GL Science) for GC-MS analysis. A standard alkane mixture (C8–C40) was injected, and the retention time for each peak of the alkane mixture was used as a reference for the retention index for tentative identification. The assigned peak intensities were normalized to the peak intensity of 2-isopropylmalic acid. Equipment conditions were adjusted according to the manufacturer’s instructions for the metabolite standard mixture.

### 2.9. Statistical Analysis

All data are presented as mean ± SD. Data were analyzed using a one-way ANOVA followed by Dunnett’s post hoc analysis (Figure 1, Figure 2, Figure 3 and Figure 5a,b,e,f), Tukey post hoc analysis (Figure 5c,d) and Student’s *t*-test (Figure 4) within Statcel-4 (OMS Inc., Saitama, Japan).

## 3. Results

### 3.1. GA Promoted the Differentiation of C2C12 Cells without Cytotoxicity

The mouse myoblast cell line, C2C12, is a widely used cell model in skeletal muscle development studies as well as age-related muscle regeneration. We first investigated the effect of GA on cell viability to determine the optimal GA concentration for use in this study. Treatment with GA (up to 0.8 mM) for 6 d did not significantly change cell viability (Figure 1a). Thus, GA did not exert any significant cytotoxic effects on C2C12 cells within the concentration range used in the subsequent experiments (0–0.8 mM).

Myosin heavy chain (MyHC) II is a representative marker of the differentiation state of myoblasts. We examined the expression level of MyHCII protein using Western blotting to evaluate the effect of GA on the differentiation of C2C12 cells. Treatment with GA for 6 d increased the expression levels of MyHCII proteins (Figure 1b). MyHCII isoforms comprise MyHCIIx, MyHCIIa, and MyHCIIb and are encoded by *Myh1*, *Myh2*, and *Myh4*, respectively. Next, we examined whether the expression of these genes is regulated by GA. As shown in Figure 1c–e, GA significantly increased the expression of these genes in a dose-dependent manner. Myoblasts in the proliferative and/or early differentiation stage express high levels of *Pax7* (a marker gene for satellite cells). *Myod1* and *Myog* are representative marker genes for the mid- and late-stage differentiation states of myoblasts, respectively [19]. The expression level of *Pax7* decreased (Figure 1f), whereas that of *Myod1* and *Myog* (Figure 1g,h) increased following treatment with GA. These results indicate that GA promotes the differentiation of C2C12 cells without exerting cytotoxic effects.

### 3.2. GA Promoted Mitochondrial Biogenesis in C2C12 Cells

Mitochondrial biogenesis is involved in regulating myogenesis and plays an important role in maintaining skeletal muscle mass and strength [20]. MtDNA copy number has been considered a well-established indicator of mitochondrial biogenesis [10], while MtMP is a key indicator of mitochondrial content because it reflects the ability of oxidative phosphorylation to generate intracellular ATP. Treatment of C2C12 cells with GA for 6 d significantly increased the mtDNA copy number (Figure 2a) and MtMP (Figure 2b) in a dose-dependent manner. These results indicate that GA increases mitochondrial content by promoting mitochondrial biogenesis. Citrate synthase (CS), a key enzyme in the first step of the tricarboxylic acid (TCA) cycle, has been used as a mitochondrial function biomarker [21]. Treatment with GA for 6 d significantly increased the mRNA (Figure 2c) and protein (Figure 2d) expression levels of CS in a dose-dependent manner. These results indicate that GA promoted mitochondrial biogenesis and increased mitochondrial content in C2C12 cells.

### 3.3. GA Increased the mRNA Levels Involved in Mitochondrial Biogenesis

Mitochondrial proteins are partially encoded by the mitochondrial genome, but most are encoded by the nuclear genome. Peroxisome proliferator-activated receptor gamma coactivator 1-alpha (PPARGC1A; also known as PGC-1α) is often described as a master regulator of mitochondrial biogenesis because it regulates mitochondrial biogenesis by regulating the transcription of nuclear-encoded mitochondrial proteins [22]. We analyzed the levels of representative mRNAs (*Sirt-1*, *Nrf1*, *Nfe2l2*, *Tfam*, and *Tfb1m*) that regulate mitochondrial biogenesis. Treatment with GA for 6 d significantly increased these mRNA levels in a dose-dependent manner (Figure 3). These results indicate that GA promotes mitochondrial biogenesis via increased expression levels of these genes.

### 3.4. GA Increased the Intracellular Levels of Several Amino Acids and Their Metabolites

The metabolism of GA and alanine to pyruvate and glycine is catalyzed by alanine:glyoxylic acid aminotransferase, mainly in the liver [23]. GA is metabolized to glycolic acid by glyoxylate reductase, which is highly expressed in the liver. However, the metabolism of GA in muscles has not been elucidated. Therefore, we investigated the metabolism of GA in muscles and the resulting metabolites in C2C12 cells using metabolome analysis. As shown in Figure 4, treatment of C2C12 cells with GA for 6 d significantly increased the intracellular levels of glycolic acid, amino acids (glycine, serine, and alanine) and their metabolites (citric acid and succinic acid). These results indicate that GA alters the profile of amino acids and their metabolites not only in the liver but also in muscle.

### 3.5. GA Suppressed Dex-Induced Increases in Muscle-Specific Ubiquitin Ligase Levels in mRNA

Dex promotes proteasome-dependent proteolysis, inhibits protein synthesis, and induces muscle atrophy [24]. MuRF1 and Atrogin1 are muscle-specific ubiquitin ligases encoded by *Trim63* and *Fbxo32*, respectively, and are recognized as representative biomarkers of muscular atrophy. Dex reportedly increases the expression of *Atrogin-1* and *MuRF1*, leading to increased ubiquitin–proteasome-dependent muscle protein degradation and decreased muscle mass [25]. Therefore, we investigated the effect of GA in a Dex-induced muscle atrophy model in C2C12 cells. GA suppressed the Dex-induced *Trim63* and *Fbxo32* expression levels in mouse-derived C2C12 cells (Figure 5a,b) and in rat-derived L6 cell (Figure 5e,f), indicating that GA suppresses Dex-induced muscle atrophy. In addition, GA also suppressed Dex-induced increases in the expression of myoatrophy markers in myoblasts during early differentiation (Figure 5c,d).

## 4. Discussion

Dietary protein intake plays a crucial role in muscle protein synthesis by increasing circulating amino acid levels [26]. Among amino acids, branched-chain amino acids (BCAAs; leucine, valine, and isoleucine) are particularly effective in stimulating muscle protein synthesis [27]. As essential amino acids, BCAAs generally cannot be synthesized in humans. However, the BCAAs produced from branched-chain keto acids (BCKAs) via the branched-chain aminotransferase reaction have the same fate as those derived from the diet [28]. BCKAs, α-keto acid metabolites from BCAAs, serve as nutrients that prevent sarcopenia in patients with CKD because they do not contain amino groups, which produce nitrogenous waste products [29]. In addition, β-hydroxy-β-methylbutyrate (HMB), a nitrogen-free metabolite derived from leucine, is effective in preventing loss of muscle mass, strength, and function in elderly individuals [30]. These findings led us to speculate that nitrogen-free metabolites have beneficial physiological effects similar to those of amino acids found in muscles. α-Keto acids are considered nutrients with low bioavailability because they are more readily metabolized than amino acids as substrates for energy metabolism [31]. Therefore, to date, a few studies have examined the functions of α-keto acids other than BCKAs in skeletal muscles.

To the best of our knowledge, this is the first study on the effects of GA, a key metabolite of glycine, on skeletal muscle. Our major findings were as follows: (i) GA promotes myoblast differentiation without cytotoxicity, (ii) GA promotes mitochondrial biogenesis via an increase in the levels of representative mRNAs (*Sirt-1*, *Nrf1*, *Nfe2l2*, *Tfam*, and *Tfb1m*) that regulate mitochondrial biogenesis, (iii) GA increases the intracellular levels of several amino acids and their metabolites, and (iv) GA ameliorates Dex-induced muscular atrophy.

Decreased muscle regeneration capacity is considered one of the principal mechanisms involved in sarcopenia. In muscle regeneration, satellite cells, which are muscle-derived stem cells, are activated and become proliferative myoblasts. Proliferating myoblasts differentiate and fuse with themselves to become multinucleated myotubes and eventually, myofibers. The decline in function of these cells contributes to the decline in the homeostasis of muscle function with aging [32]. Therefore, myoblast differentiation plays an important role in maintaining the homeostasis of muscle function. In the present study, Western blot analysis and qPCR showed that both MyHCII protein and mRNA levels increased with GA treatment. Furthermore, the expression level of *Pax7* decreased, whereas that of *Myod1* and *Myog* increased with GA treatment. Myoblast differentiation is regulated by myogenic regulatory factors (MRFs), such as MyoG and MyoD, which are encoded by *Myog* and *Myod1*, respectively [33]. It is well known that MyoD regulates the expression of Myog and functions as a master regulator of differentiation in C2C12 cells [34]. The overexpression of *Pax*, a satellite cell marker, reduces *Myod1* and *Myog* expression, resulting in the inhibition of myoblast differentiation [35]. In the present study, GA induced a decrease in *Pax7* expression and an increase in *Myod1* and *Myog* expression. These results suggest that GA might contribute to myogenesis through the differentiation of C2C12 cells.

Aging is associated with mitochondrial dysfunction, which leads to a decline in the quality and quantity of skeletal muscle [36]. Maintaining the mitochondrial redox environment prevents a decline in mtDNA abundance and mitochondrial biogenesis in the muscles of aged mice [37]. Reactive oxygen species (ROS) generated in the mitochondrial electron transport chain induce intracellular oxidative damage and lead to an age-related decline in skeletal muscle [38]. GA has antioxidant properties owing to its aldehyde group. Therefore, we examined the physiological effects of GA on mitochondrial function. As expected, GA increased the mtDNA copy number, MtMP, and the expression level of CS in C2C12 cells. Compared with myogenic precursor cells, differentiated myofibers have a higher mitochondrial function consisting of increased mtDNA, MtMP, and expression levels of TCA cycle enzymes to meet elevated energy demands [39]. These results suggest that GA may promote myoblast differentiation by meeting energy demands by promoting mitochondrial biogenesis.

Calorie restriction and moderate exercise are well-known strategies for promoting mitochondrial biogenesis. Notably, nutritional supplementation with a combination of essential amino acids and branched-chain amino acids promoted mitochondrial biogenesis and prevented deterioration of muscle function in an aging mouse model [40]. In addition, leucine induces mitochondrial biogenesis in muscle cells by stimulating the expression of PGC-1α and NRF-1 via a SIRT1-dependent pathway in muscle cells [41]. Mitochondrial biogenesis is regulated by mtDNA replication and the expression of mitochondrial and nuclear genes such as *Sirt1*, *ppargc1a*, *Nrf1*, *Nfe2l2*, *Tfam*, and *Tfb1m* [42]. The present study revealed that GA promotes mitochondrial biogenesis via increased expression levels of these genes in C2C12 cells.

Transamination is a well-known metabolic reaction in which amino groups are transferred from amino acids to α-keto acids, and it is particularly active in the liver, skeletal and heart muscle, and kidney. In the liver, the metabolism of alanine and GA to pyruvic acid and glycine, respectively, is catalyzed by alanine:glyoxylic acid aminotransferase [23]. To the best of our knowledge, this is the first study to show that GA alters the profile of amino acids and their metabolites in the muscle cells. Treatment of C2C12 cells with GA for 6 d significantly increased the intracellular levels of glycolic acid, amino acids (glycine, serine, and alanine), and their metabolites (citric acid and succinic acid) in this study. Notably, these increases were observed in the metabolites in a pathway closely associated with GA. If the increase in glycine content resulting from GA treatment is primarily due to alanine:glyoxylic acid aminotransferase catalysis, an intracellular decrease in alanine level and an increase in pyruvic acid level might be observed. However, the intracellular alanine content was increased by GA treatment, whereas pyruvic acid was insensitive and did not yield reliable intracellular quantification results. These results made it difficult to determine the source of the amino groups donated to GA. In addition, the expression level of CS, a biomarker of mitochondrial content, increased upon GA treatment. This enzyme catalyzes the condensation of acetyl-CoA and oxaloacetic acid to form citric acid. It has been suggested that the activation of CS in the mitochondria and an increase in intracellular substrates are involved in the increase in citric acid level. DMEM contains amino acids such as glycine and serine, which are sources of GA. Thus, GA may be synthesized intracellularly from these amino acids, although it is not included in the medium. It would be important to use media that do not contain these amino acids or alanine:glyoxylic acid aminotransferase-knockdown cells to further clarify the mechanism of action of GA.

Interestingly, a decrease in the level of skeletal muscle glycine is prominent in mouse models of muscular dystrophy and frail elderly individuals [43,44]. Under these conditions, the demand for glycine may be increased due to the inactivation of synthetic pathways or activation of degradation pathways in the metabolic pathways of glycine in the muscle. In addition, glycine decreases the rate of protein degradation by regulating muscle-specific ubiquitin ligase expression in C2C12 cells [45]. Using metabolomic analysis, we showed that GA is metabolized to glycine. Therefore, we measured the expression levels of *Trim63* and *Fbxo32* in a Dex-induced muscular atrophy model to investigate whether GA suppresses muscular atrophy in skeletal muscle cells. As expected, GA suppressed the Dex-induced increase in the expression of *Trim63* and *Fbxo32*, muscle-specific ubiquitin ligase genes involved in muscular atrophy. These results suggest that GA may not only exhibit these effects on its own, but also function as a source of glycine. Recently, it has been shown that microgravity inhibits C2C12 cell differentiation via changes in calcium homeostasis [46]. Calcium is strongly involved in muscle contraction and is therefore essential for the maintenance of muscle and bone functions. Therefore, to elucidate the effects of amino acids and keto acids on the decline in muscle function with aging, changes in calcium homeostasis should also be examined.

Currently, GA may be regarded as a substance that induces ureteral stones. Therefore, further in vivo experiments should be performed to not only clarify the physiological effects of GA as a source of amino acids, but also determine whether GA produces oxalates in the toxic range and whether it is a safe nutrient for individuals with low waste excretion and limited nitrogen intake.

## 5. Conclusions

The results presented herein showed that GA promotes differentiation and mitochondrial biogenesis and ameliorates Dex-induced muscle atrophy in C2C12 cells. In addition, this is the first study to show that GA alters the profile of amino acids and their metabolites in muscle cells. In addition, it would be important to determine the effects of long-term oral intake of GA on muscle mass and strength.

After evaluating these experiments, intervention studies in CKD patients undergoing strict dietary protein restriction should demonstrate whether GA has an effect on improving loss of muscle mass, strength, and function. Currently, GA may be regarded as a substance that induces ureteral stones; however, in the future, it may be an important nutrient for promoting myogenesis in skeletal muscle, similar to keto acid analogs derived from essential amino acids without producing urinary toxins.

## Data Availability

Data from the present study are not publicly available. Data are available upon reasonable request and with permission from the authors.

## Supplementary Materials

The following supporting information can be downloaded at: https://www.mdpi.com/article/10.3390/nu15071763/s1, Table S1: Primers used for quantitative real-time polymerase chain reaction (qRT-PCR). Figure S1: Melt curve analysis of quantitative real-time polymerase chain reaction products by SYBR Green assay.
