# Supplementary material for: Glyoxylic Acid, an α-Keto Acid Metabolite Derived from Glycine, Promotes Myogenesis in C2C12 Cells
Source: Nutrients. 2023 Apr 4;15(7):1763. doi: 10.3390/nu15071763 (PMC10096605; doi:10.3390/nu15071763)
Supplement: Supplementary file 1 [file nutrients-15-01763-s001.zip › nutrients-2330941-supplementary.pdf]

Table S1: Primers used for quantitative real-time polymerase chain reaction (qRT-PCR)

Name (organism)	Sequence (5'-3') (F, forward; R, reverse)	GeneBank No.	Product size (bp)
Myh1 *(mouse)*	F: AAAGGCAGGCTCTCTCACTGG<br>R: CCTCGATTCGCTCCTTTTCAG	NM_030679.2	123
Myh2 *(mouse)*	F: TGAGGAGGCTGAGGAACAATC<br>R: AGCATCGGGACAGCCTTACT	NM_001039545.2	178
Myh4 *(mouse)*	F: CTTCGTAAGCACGAGCGCA<br>R: TTGGATTGTTCCTCAGCCTCCT	NM_010855.3	152
Pax7 *(mouse)*	F: TGAGTTCGATTAGCCGAGTGC<br>R: CCTCATCCAGACGGTTCCCT	NM_011039.2	147
Myod1 *(mouse)*	F: ATGGTGTCCCTGGTTCTTCAC<br>R: GACTTCTGCTCTTCCCTTCCC	NM_010866.2	105
Myog *(mouse)*	F: CCCAACCCAGGAGATCATTTG<br>R: CAGTTGGGCATGGTTTCGTC	NM_031189.2	134
Actb *(mouse)*	F: CATCCGTAAAGACCTCTATGCCAAC<br>R: ATGGAGCCACCGATCCACA	NM_007393.5	171
16S *(mouse)*	F: CCGCAAGGGAAAGATGAAAGAC<br>R: TCGTTTGGTTTCGGGGTTTC	NC_005089.1	133
Actb *(mouse)*	F: CATCCGTAAAGACCTCTATGCCAAC<br>R: ATGGAGCCACCGATCCACA	NC_000071.7	296
Cs *(mouse)*	F: CCATGTTCAAGCTGGTGGCT<br>R: TCCGTCATGCCATAGTACTGGAG	NM_026444.4	133
Sirt1 *(mouse)*	F: GGTAGTTCCTCGGTGCCCTA<br>R: ACCAAAGAAGACAATCTCTGGCT	NM_019812.3	76
Ppargc1a *(mouse)*	F: ACCACAAACGATGACCCTCC<br>R: GTTGGTTTGGCTTGAGCATGT	NM_008904.2	125
Nrf1 *(mouse)*	F: TCTCACCCTCCAAACCCAAC<br>R: GCGGCAGCTCTGAATTAACC	NM_001361693.1	156
Nfe2l2 *(mouse)*	F: CTCCCAGGTTGCCCACATTC<br>R: TATCCAGGGCAAGCGACTCA	NM_010902.4	113
Tfam *(mouse)*	F: CCGTATTGCGTGAGACGAAC<br>R: GACAAGACTGATAGACGAGGGGA	NM_009360.4	164
Tfb1m *(mouse)*	F: TACGCCCTTGATAGAGCCCAA<br>R: AACATTCCAAGCCCTCGGTG	NM_146074.3	111
Trim63 *(mouse)*	F: CTGAGTAACTGCATCTCCATGCT<br>R: CTGGTGGCTATTCTCCTTGGT	NM_001369245.1	105
Fbxo32 *(mouse)*	F: GTCTTTTGGCAGTGAGCCCT<br>R: TATCACCACCCCTTGCCTTAC	NM_026346.3	134
Trim63 *(Rat)*	F: CAGTACCGAGAGCAGTTGGAA<br>R: GCACTCAAGAGGAAGGTGGC	NM_080903.2	95
Fbxo32 *(Rat)*	F: ACATGTGGGTGTATCGAATGG<br>R: AGCCTCTGCATGATGTTCAGT	NM_133521.2	139
Actb *(Rat)*	F: GGAGATTACTGCCCTGGCTCCTA<br>R: GACTCATCGTACTCCTGCTTGCTG	NM_031144.3	150

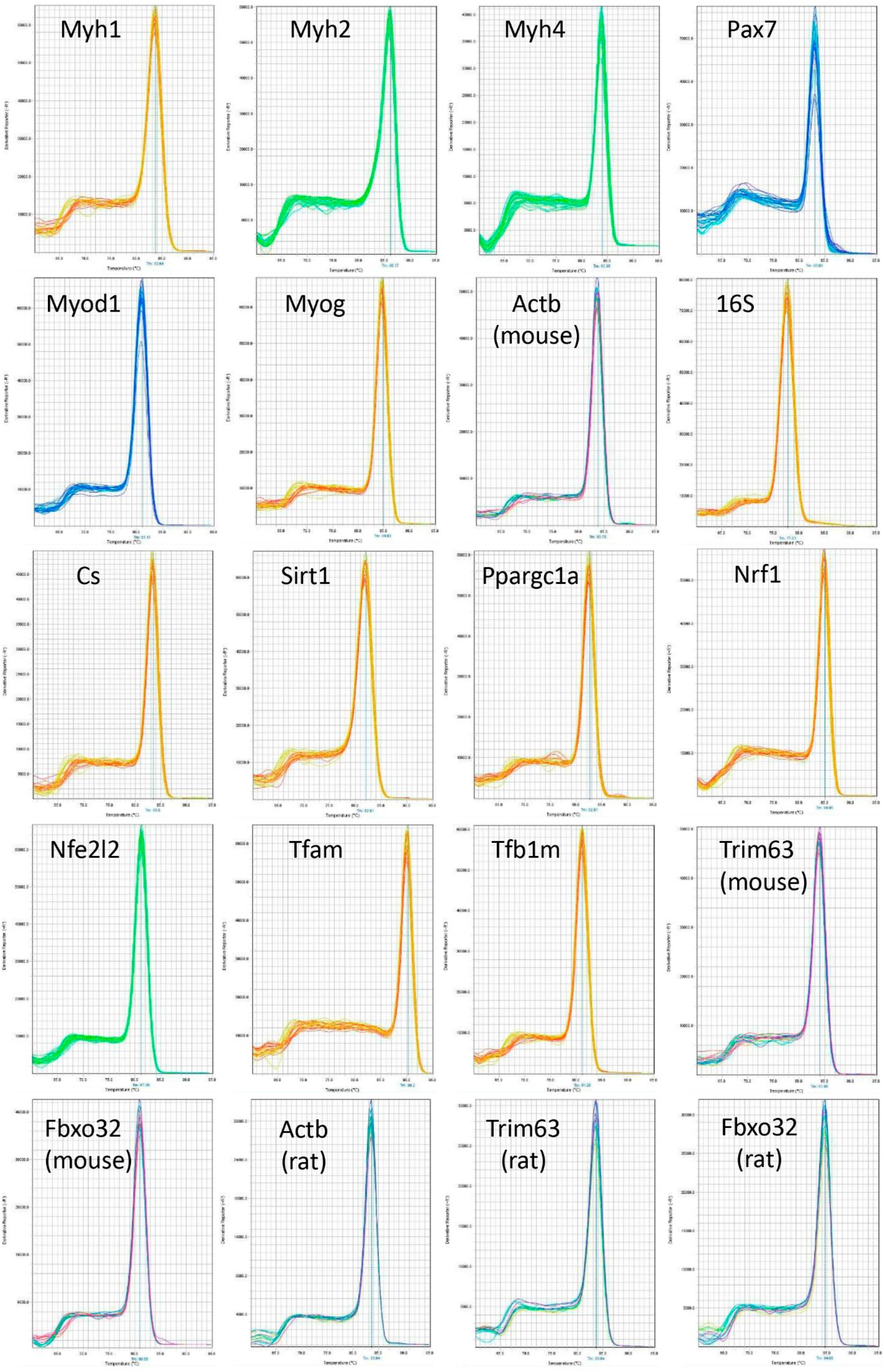

Figure S1 Melt curve analysis of quantitative real-time polymerase chain reaction products by SYBR Green assay
